# Supplementary material for: Comparative transcriptomic analyses and single-cell RNA sequencing of the freshwater planarian Schmidtea mediterranea identify major cell types and pathway conservation
Source: Genome Biol. 2018 Aug 24;19:124. doi: 10.1186/s13059-018-1498-x (PMC6109357; doi:10.1186/s13059-018-1498-x)
Supplement: Supplementary file 2 — Figure S1. Comparative analysis of the mapped and unmapped transcripts of Toronto and PlanMine transcriptomes onto dd_Smes_g4 genome assembly. Figure S2. Phylogenetic profiling of S. mediterranea based on GO Slim terms. Piecharts showing phylogenetic breakdown of various GO Slim groupings. Figure S3. Phylogenetic distribution of cadherins from human, C. elegans, Platyhelminthes, and S. mediterranea. Phylogenetic tree of cadherins. Figure S4. Gene expression profiles of Cluster 7 subclusters. Figure S5. Smed-egr5 gene expression and phenotypes. (PDF 1428 kb) [file 13059_2018_1498_MOESM2_ESM.pdf]

# Comparative transcriptomic analyses and single-cell RNA sequencing of the freshwater planarian *Schmidtea mediterranea* identifies major cell types and pathway conservation

## Supplementary Figures

**Figure S1:** Comparative analysis of the mapped and unmapped transcripts of Toronto and Planmine transcriptomes onto dd_Smes_g4 genome assembly

**Figure S2:** Phylogenetic profiling of *S. mediterranea* based on GOSlim terms. Piecharts showing phylogenetic breakdown of various GOSlim groupings

**Figure S3:** Phylogenetic distribution of cadherins from human, *C. elegans*, platyhelminthes, and *S. mediterranea*. Phylogenetic tree of cadherins

**Figure S4:** Gene expression profiles of Cluster 7 sub-clusters

**Figure S5:** Smed-egr5 gene expression and phenotypes

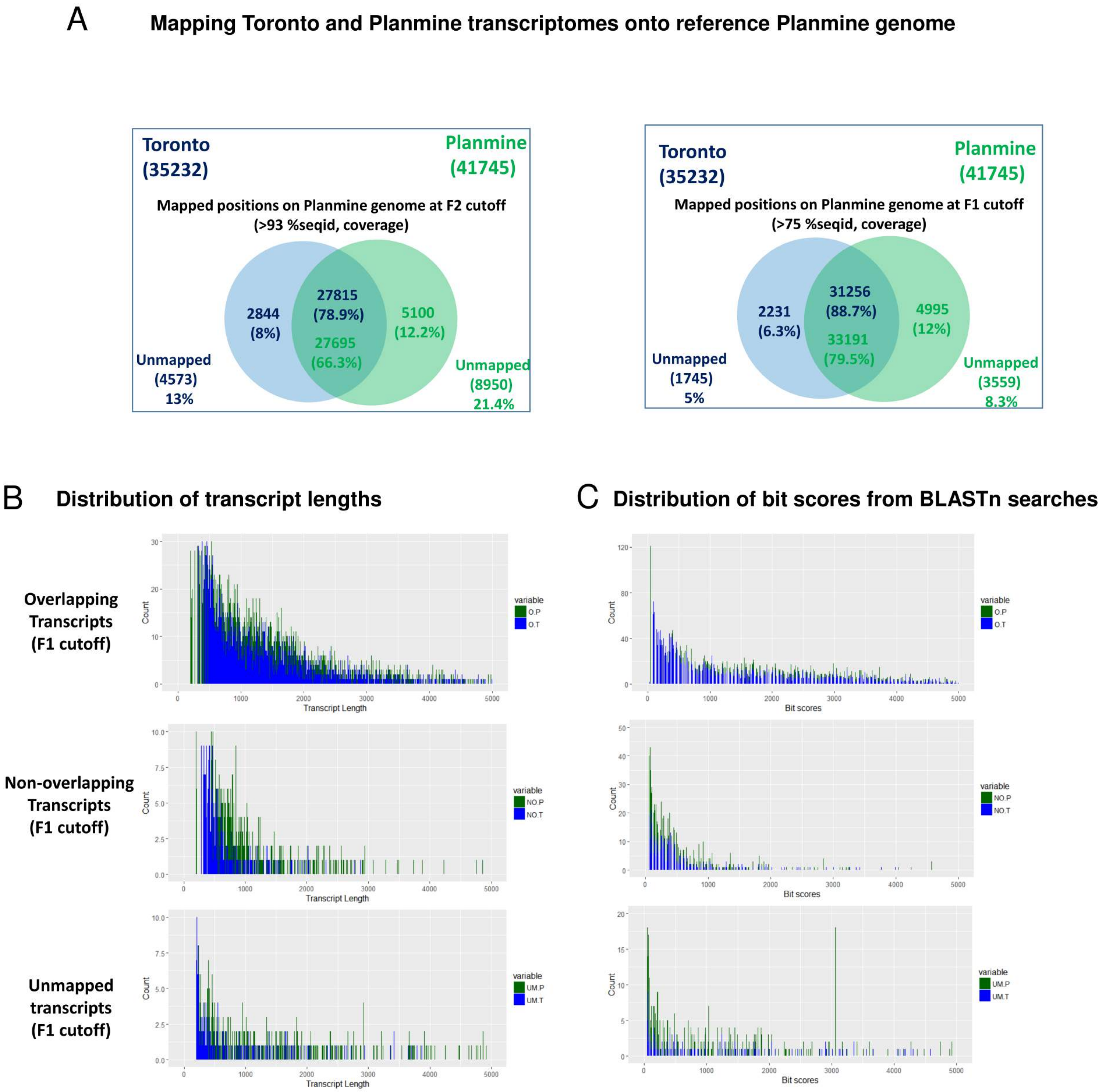


**Figure S1:** Comparative analysis of the mapped and unmapped transcripts of Toronto and Planmine transcriptomes onto dd_Smes_g4 genome assembly. A) Venn diagrams showing the distribution of transcripts from Toronto and Planmine transcriptomes mapped (overlapping, non-overlapping) as well as unmapped on the genome using SPALN at strict (F2) and mild (F1) cutoffs. Distribution of B) transcripts lengths and C) bit scores from BLASTn searches for mapped (overlapping, non-overlapping) and unmapped transcripts. Legend keys: O.P - Overlapping-Planmine, O.T - Overlapping.Toronto, NO.P - Nonoverlapping-Planmine, NO.T - Nonoverlapping.Toronto, UM.P - Unmapped Planmine, UM.T - Unmapped.Toronto.

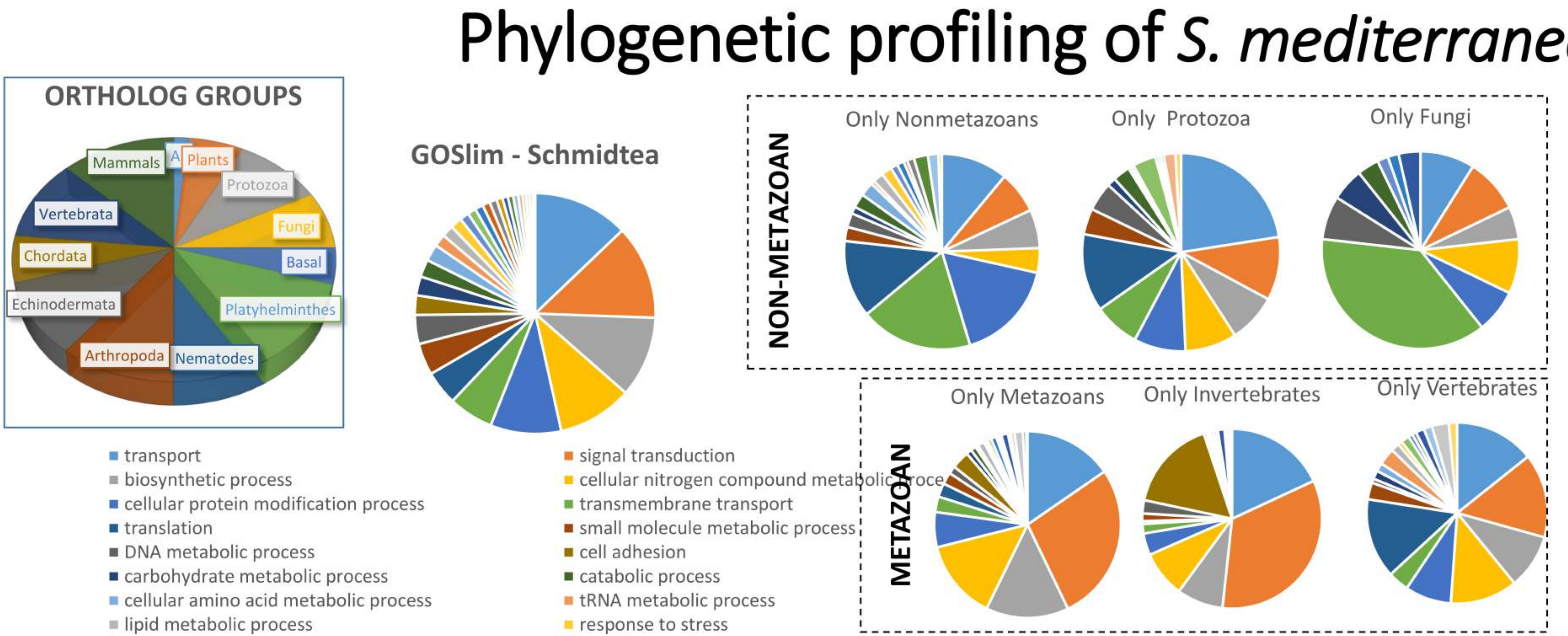


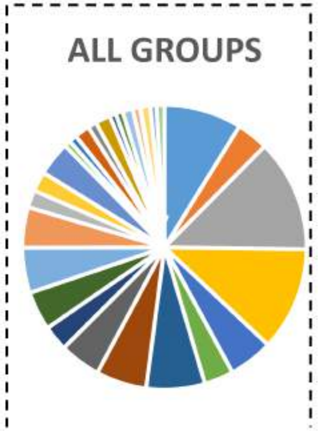


**Figure S2:** Phylogenetic profiling of *S. mediterranea* transcripts based on GOSlim terms mapped via Interpro2GO mappings. Piecharts showing breakdown of various GOSlim mappings for the different taxonomic groups.

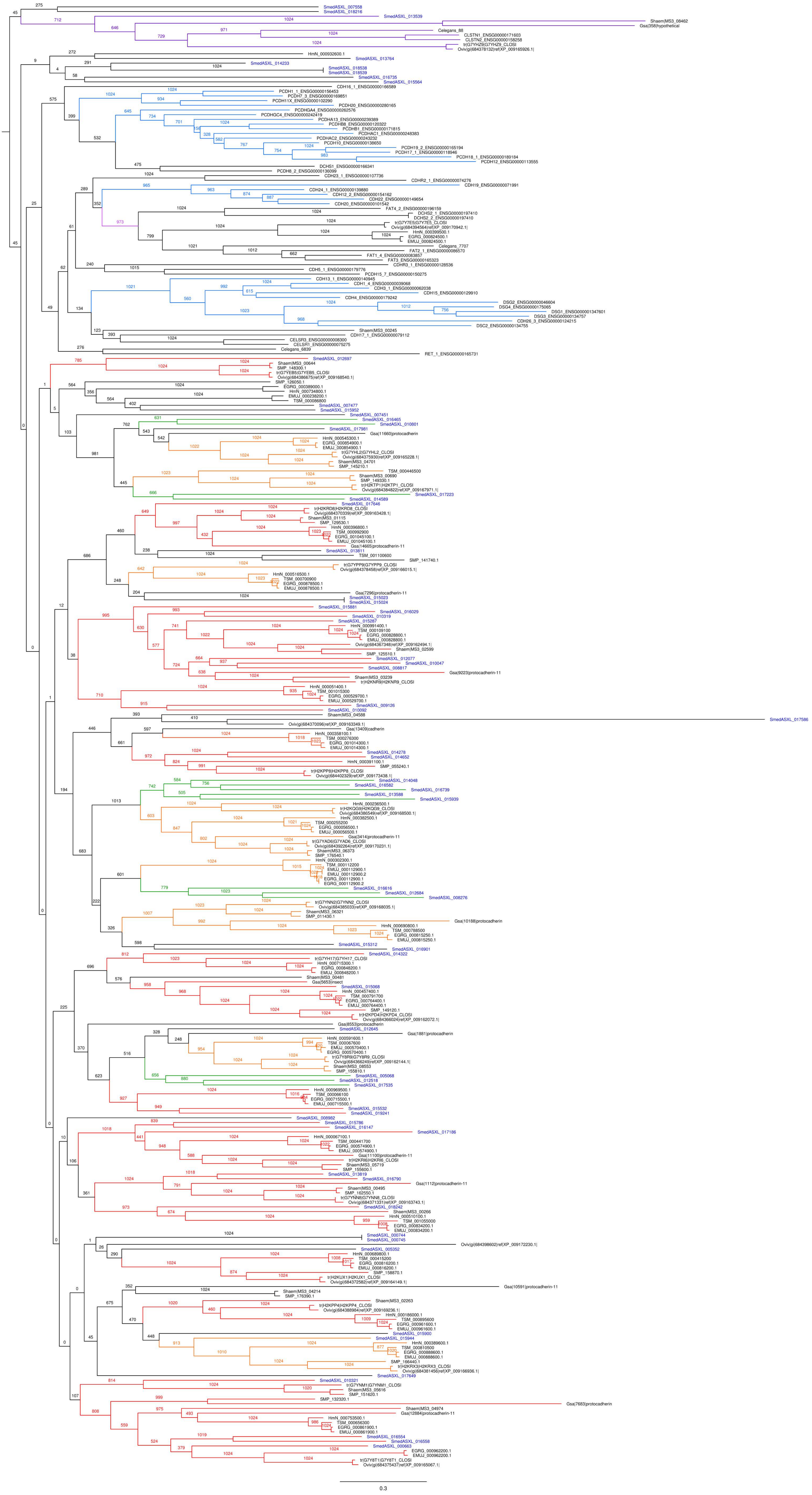

**Figure S3:** Phylogenetic distribution of cadherins from human, *C. elegans*, worms (platyhelmenthes), and *S. mediterranea*. Clades with bootstrap support of >600/1000 are colored by the taxonomic representation of the species in each clade: Light blue - human, Purple - human + worms + *S. mediterranea*, Pink - human + worms, Orange - worms, Red - worms + *S. mediterranea*, Green - *S. mediterranea-specific*.

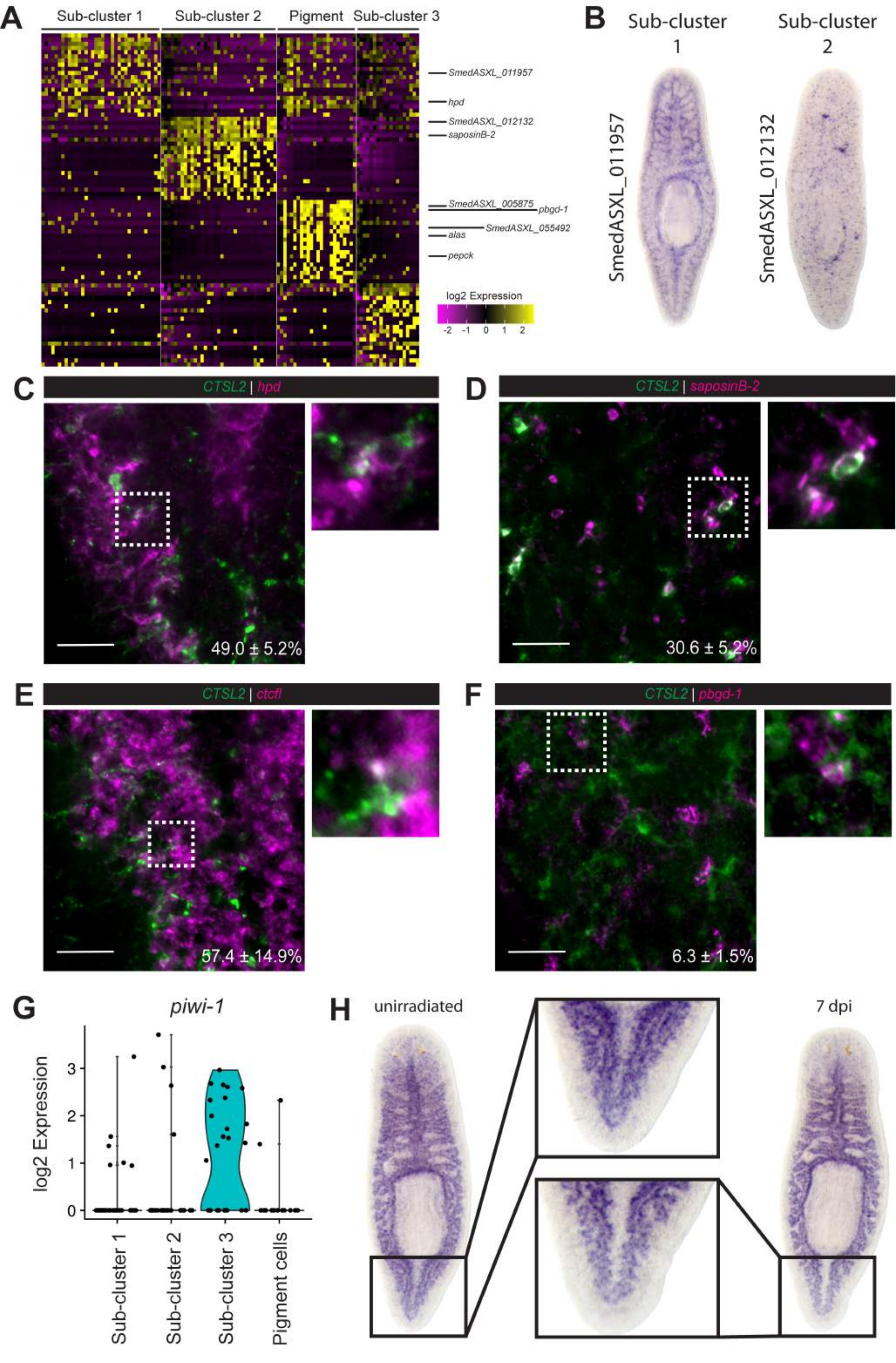


**Figure S4:** Cluster 7 sub-clusters have distinct gene expression profiles. A) Heatmap of the top 20 genes enriched in each sub-cluster identified by Seurat. B) Whole-mount in situ hybridizations of additional sub-cluster markers. C-F) dFISH of Smed-CTSL2 with sub-cluster 1 marker hpd (C), sub-cluster 2 marker saposinB-2 (D), sub-cluster 3 marker ctcfl (E) and pigment cell marker pbgd-1 (F). Zoomed images of the boxed areas are shown in panels on the right. % overlap of sub-cluster markers with Smed-CTSL2 is indicated (n = 3-4). Images are 3 um confocal z-stacks. Scale bars = 50 um. G) Violin plot of piwi-1 expression demonstrating enrichment in Sub-cluster 3. H) Whole-mount in situ hybridizations of the sub-cluster 3 marker ctcfl in unirradiated and lethally irradiated worms (7 days post-irradiation). Zoomed in images of the tail regions demonstrate loss of ctcfl expression in the mesenchymal area between the posterior gut branches at 7dpi.

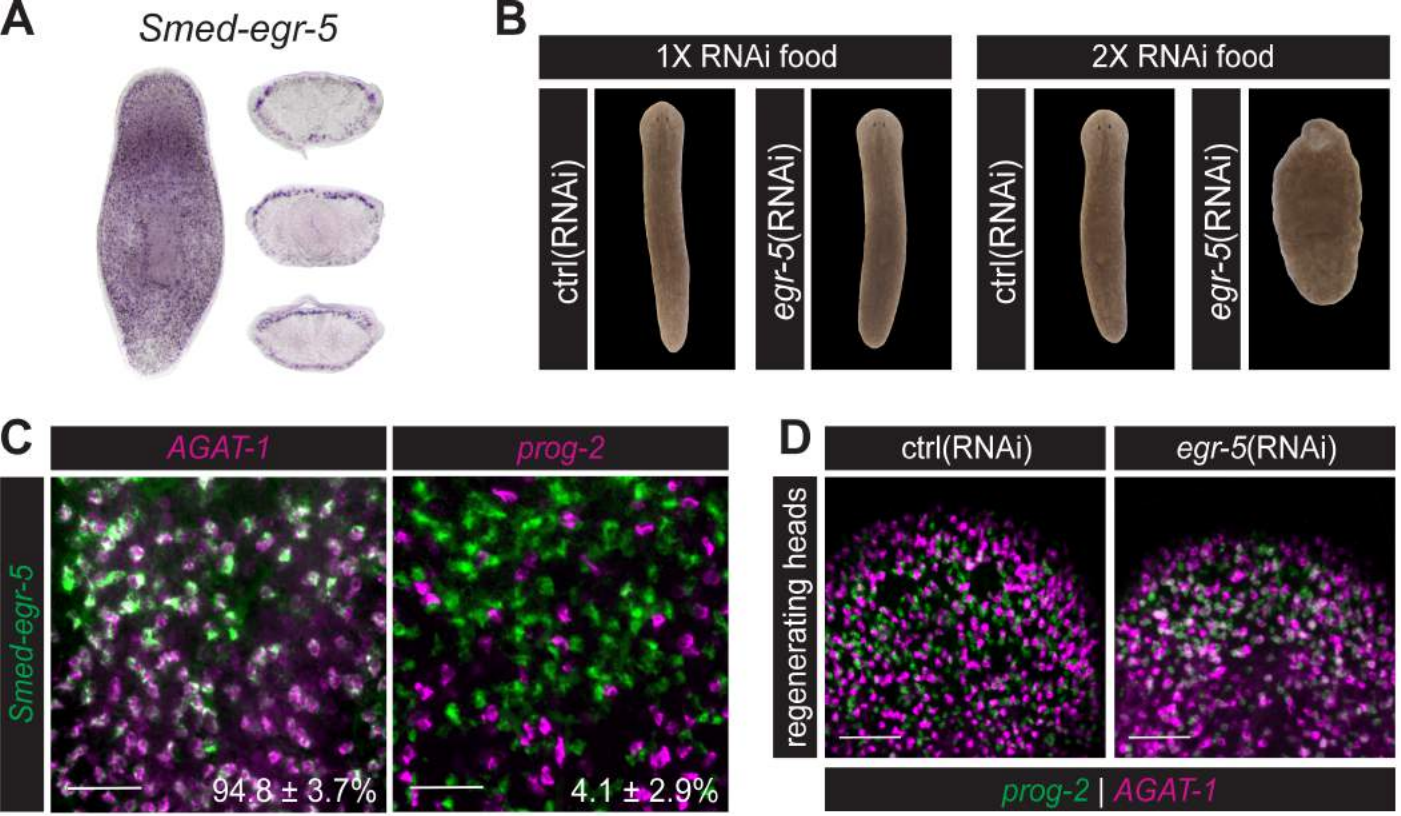


**Figure S5:** A) in situ hybridization of Smed-egr-5 in whole-mount and transverse cross-sections. B) Brightfield images of Smed-egr-5(RNAi) animals at 6fd10. Animals fed 2X RNAi food exhibited tissue regression and lysis, as previously reported. Animals fed 1X RNAi food did not exhibit any homeostatic phenotype. n = 10. C) dFISH of Smed-egr-5 demonstrates that the majority of Smed-egr-5+ cells express the late epithelial progenitor marker AGAT-1, but not the early epithelial progenitor marker prog-2. Images are single confocal slices taken from the tail. Scale bars = 50 um. n = 4-5. D) No striking defects in epithelial regeneration were observed following knockdown of Smed-egr-5 with 1X RNAi food in regenerating heads. Images are single confocal slices taken from regenerating head tissue. Scale bars = 50 um. n = 10.
